# Repurposing Metformin in Precision Oncology: Mechanistic Insights, Biomarker-Guided Strategies, and Translational Imperatives

**DOI:** 10.3390/medicina61091577

**Published:** 2025-08-31

**Authors:** Shehla Shafi Khan, Imran Rashid Rangraze, Adil Farooq Wali, Malay Jhancy, Rasha Aziz Attia, Hesham Amin Hamdy Elshamly, Shukri Adam, Rana Aly Mohamed Elbeshbeishy

**Affiliations:** 1 RAK College of Medical Sciences, RAK Medical and Health Sciences University, Ras Al Khaimah P.O. Box 11172, United Arab Emirates; 2 RAK College of Pharmacy, Ras Al Khaimah Medical and Health Sciences University, Ras Al Khaimah P.O. Box 11172, United Arab Emirates; 3 RAK College of Nursing, Ras Al Khaimah Medical and Health Sciences University, Ras Al Khaimah P.O. Box 11172, United Arab Emirates

**Keywords:** metformin, precision oncology, anticancer mechanisms, biomarkers, AMPK pathway, tumor heterogeneity, cancer stem cells, drug repurposing, translational oncology

## Abstract

*Background and Objectives*: Metformin, a staple in diabetes care, has recently emerged as a candidate chemotherapeutic agent. In vitro studies suggest that metformin inhibits cancer growth by altering cellular metabolism and enhancing immune responses. Clinical observations further indicate that it suppresses key tumor-promoting pathways such as mTOR and STAT3. This review critically evaluates the therapeutic potential of metformin in oncology through the lens of precision medicine. This review integrates evidence from molecular mechanisms, clinical studies, biomarker-driven trial designs, and the regulatory challenges that continue to delay its approval for oncologic use. *Methods*: A structured literature search (2015–2025) identified 63 relevant studies, including preclinical, clinical, and translational research. *Conclusions*: Although metformin shows consistent anticancer effects in laboratory and animal models, its clinical benefits in patients are variable. This inconsistency is likely due to tumor heterogeneity and a lack of biomarker-based patient selection in trials. Targeting these shortcomings through biomarker-enriched, tumor-specific clinical trials is essential to define metformin’s role as a repurposed agent in precision oncology.

## 1. Introduction

Cancer therapy has shifted dramatically from conventional one-size-fits-all regimens toward precision medicine, where interventions are guided by the molecular, genetic, and metabolic characteristics of each tumor. This evolution underscores the importance of cost-effective, safe, and widely accessible agents that can complement or enhance existing therapies. Within this context, metformin—a first-line treatment for type 2 diabetes—has emerged as a promising candidate for drug repurposing in oncology. Its pleiotropic effects on cellular metabolism, oncogenic signaling, and the tumor microenvironment highlight its potential to serve as an adjunct in precision oncology [1,2].

### 1.1. Precision Oncology: A Paradigm Shift in Cancer Therapy

Precision oncology employs high-throughput sequencing, multi-omics platforms, and biomarker discovery to guide personalized treatment strategies. Molecular tumor boards increasingly integrate genomic data, pathway alterations, and predictive biomarkers to identify actionable targets. While these advances have transformed care, the high cost and infrastructure demands of recently approved targeted therapies often limit patient access—particularly in low- and middle-income countries—making affordable alternatives essential [3].

### 1.2. Metformin in Oncology: A Repurposed Candidate

Beyond glycemic control, metformin modulates the key biological pathways implicated in cancer progression. It activates AMP-activated protein kinase (AMPK), inhibits mTOR signaling, and attenuates insulin/IGF-1-mediated oncogenic cascades. Experimental studies demonstrate effects on cancer stem cells, angiogenesis, and immune surveillance. Observational and early clinical data further suggest survival benefits in selected populations, supporting its consideration as a low-cost, repurposed anticancer therapy.

### 1.3. The Role of Biomarker-Guided Strategies

Patient responses to metformin in oncology remain inconsistent, reflecting tumor heterogeneity, diverse metabolic profiles, and genetic variability. Biomarker-guided approaches—including genomic alterations (e.g., PI3K/AKT/mTOR pathway mutations), transporter polymorphisms, metabolomic signatures, and advanced imaging—can refine patient selection. Such strategies hold promise for identifying responsive subgroups and optimizing trial designs, moving metformin closer to routine clinical application in precision oncology [3].

### 1.4. Rationale and Scope of This Review

This review synthesizes evidence on the mechanistic foundations of metformin’s anticancer activity, its preclinical and clinical performance, and the emerging role played by biomarker-guided strategies in patient stratification [4,5,6]. It also examines pharmacological, regulatory, and ethical considerations associated with its repurposing. By integrating mechanistic insights with translational and clinical perspectives, this article aims to clarify the opportunities and challenges in establishing metformin as a validated component of precision oncology.

To understand the clinical implications of metformin in oncology, it is first essential to examine the underlying biological mechanisms that explain its potential anticancer activity. Therefore, the following section outlines the mechanistic foundations of metformin’s action beyond glycemic control, providing a scientific basis for later discussions on preclinical and clinical evidence.

## 2. Methodology

A structured search of PubMed, Scopus, and Web of Science (2015–2025) identified 63 eligible peer-reviewed articles after relevance screening and duplicate removal. The evidence was synthesized across six domains: molecular mechanisms, preclinical and clinical findings, predictive biomarkers, pharmacological and resistance considerations, regulatory and ethical aspects, and future perspectives.

## 3. Mechanistic Insights: Metformin’s Anticancer Action Beyond Glycemic Control

Mechanistic insights into metformin’s actions provide the rationale for its repurposing in oncology. By modulating cellular energy pathways, oncogenic signaling, and the tumor microenvironment, metformin exerts effects that extend well beyond glucose regulation.

Metformin is most familiar as a first-line medication for type 2 diabetes, yet investigators have begun to spotlight it as a potential anticancer drug because it redraws several critical maps of cell metabolism and signaling. Population-based studies, including large cohorts of patients with type 2 diabetes, link the medication to lower cancer rates and longer survival after diagnosis [4,5,6].

However, the clinical findings remain inconsistent. Outcome variability stems from the tumor’s biological diversity and a lack of validated selection biomarkers, underscoring the urgent need for studies that enroll patients on the basis of specific tumor features. Consequently, metformin stands at a pivotal point in translation, and its place in precision oncology depends on research that couples clear mechanistic insight with robust patient stratification [7].

### 3.1. Targeting Cellular Metabolism

Metformin primarily acts by inhibiting mitochondrial complex I of the electron transport chain, leading to a drop in ATP production and activation of AMP-activated protein kinase (AMPK), a master regulator of cellular energy homeostasis [8]. This shift favors catabolic over anabolic processes, reducing tumor-promoting activities such as protein and lipid synthesis while enhancing autophagy and oxidative stress sensitivity [9] (Table 1).

**Figure 1:**
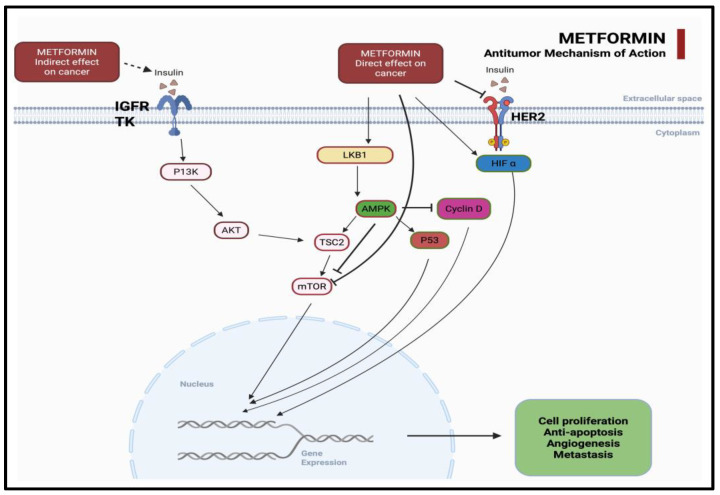
Antitumor mechanism of metformin. Metformin exerts indirect effects by lowering insulin and suppressing the IGFR-TK–PI3K–AKT–mTOR pathway. Directly, it activates LKB1–AMPK, inhibits TSC2, mTOR, HIF-α, and Cyclin D, and enhances P53, leading to reduced cell proliferation, angiogenesis, metastasis, and anti-apoptotic signaling.

In hepatocytes and tumor cells alike, metformin reduces hepatic gluconeogenesis and systemic insulin levels, thereby blunting the insulin/IGF-1 signaling cascade implicated in tumorigenesis [10]. Moreover, by disrupting the Warburg effect and restoring oxidative metabolism, metformin can deprive tumor cells of their preferred energy source [11] (Figure 1).

### 3.2. Modulation of Oncogenic Signaling Pathways

AMPK activation results in mTOR inhibition, which plays a central node in the regulation of cell proliferation and survival [12]. Metformin also interferes with mitogenic signaling via the attenuation of PI3K/AKT and IRS-1 activity, leading to reduced downstream oncogenic activity. Furthermore, the suppression of NF-κB and COX-2 dampens tumor-associated inflammation.

Recent work indicates that mTOR is not the sole target of metformin in oncology [13]. The drug also appears to dampen cancer stemness by suppressing TGF-β activity and by lowering Snail and Notch pathway output, steps that drive epithelial-to-mesenchymal transition, or EMT, and subsequent metastatic spread [14].

### 3.3. Influence on Cancer Stem Cell Plasticity and Mitochondrial Biogenesis

Recent preclinical observations suggest that metformin exerts selective toxicity toward cancer stem cells (CSCs), the resilient minority often blamed for tumor onset, recurrence, and therapeutic escape [15]. In laboratory models of breast carcinoma, ovarian carcinoma, and glioblastoma, the drug compromises CSC survival by disrupting mitochondrial oxidative phosphorylation and curbing the expression of core pluripotency regulators such as OCT4 and Nanog [16].

Recent work indicates that metformin promotes mitochondrial turnover, forcing certain cancer cells into an acute energy deficit. By simultaneously throttling glycolytic flux and inhibiting oxidative phosphorylation, the drug exposes cancer stem cells to remarkable stress, a vulnerability that is further magnified in hypoxic or nutrient-poor microenvironments [17,18,19].

Nevertheless, a growing body of literature documents context-dependent resistance in cancer stem cells, with several populations exhibiting metabolic plasticity that renders them resilient to metformin [20]. Notably, heightened glutaminolysis flux or increased reliance on fatty acid oxidation frequently underpins this adaptive survival. Such observations have prompted researchers to pair the biguanide with pharmacological blockades of these compensatory routes in hopes of enhancing overall cytotoxic potency [21,22].

### 3.4. Reprogramming the Tumor Microenvironment

**Figure 2:**
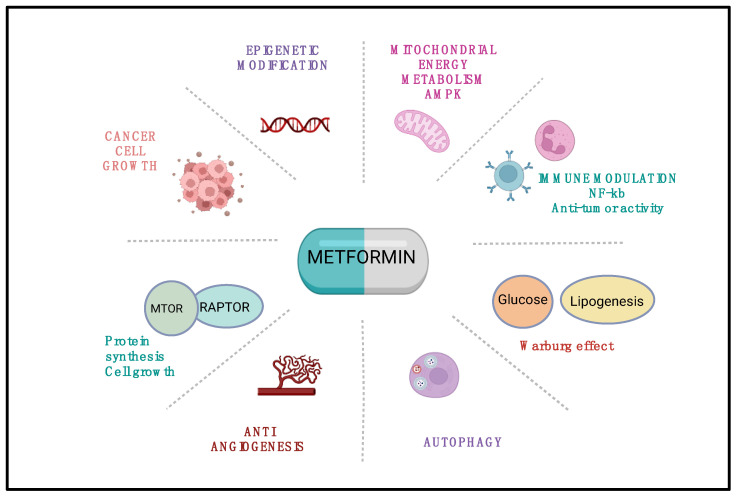
The effects of metformin on cellular metabolism, signaling pathways, and tumor microenvironment. NF-κB—nuclear factor kappa light chain-enhancer of activated B cells.

Metformin remodels the tumor microenvironment by reducing fibrosis and lowering extracellular matrix stiffness through the inhibition of activated cancer-associated fibroblasts. It also enhances antitumor immunity by provoking cytotoxic T cells and natural killer (NK) cells while tempering immunosuppressive populations, notably regulatory T cells (Tregs) and myeloid-derived suppressor cells (MDSCs). In addition, the drug promotes the normalization of aberrant tumor blood vessels, thereby improving the delivery of co-administered therapeutics to previously poorly perfused regions (Figure 2) [23,24,25].

A number of in vitro and animal experiments have highlighted pathways such as AMPK activation and mTOR suppression, yet the jump from bench to bedside remains precarious. Cell culture studies frequently spike metformin to 1–10 mM, doses that dwarf the 10–50 µM found in human plasma after ordinary prescriptions. Any observed cytotoxic or metabolic shock effect at those concentrations cannot be assumed to mirror what occurs in living patients. Adding to the uncertainty, standard two-dimensional cultures ignore the hypoxia, drug gradients, and mixed metabolite pools that characterize a native tumor microenvironment, so the broad applicability of these preclinical observations is open to debate [12,26].

## 4. Preclinical Evidence Supporting Metformin’s Anticancer Effects

Preclinical research that spans in vitro cell cultures, in vivo rodent models, and detailed mechanistic probing has begun to paint metformin as a candidate agent against several tumor types. Investigators have observed that the drug can stifle cell division, trigger apoptotic pathways, block the formation of new blood vessels, and undermine the viability of putative cancer stem cells. Table 2 provides a summary of representative preclinical investigations.

Table 2 provides a concise overview of the key findings from in vitro studies, in vivo studies, and mechanistic insights derived from animal models regarding metformin’s anticancer properties.

Multiple preclinical investigations report that metformin arrests tumor cell growth, triggers programmed cell death, and boosts the vulnerability of neoplasms to both chemotherapeutic agents and ionizing radiation. Similar findings are detailed in experimental models of breast, prostate, colorectal, and lung malignancies.

Preclinical studies consistently observe metformin’s bioenergetic effects, yet many experiments employ culture media spiked with millimolar drug levels. Murine dosing regimens, even when converted to human equivalents, frequently exceed routine clinical pharmacokinetics. Collectively, those discrepancies urge restraint when projecting efficacy into patient care [35,36,37].

## 5. Clinical Evidence and Observational Studies

Recent clinical research, from large epidemiological surveys to smaller retrospective cohorts and more rigorously designed randomized controlled trials (RCTs), has begun to scrutinize metformin in the oncology setting [38]. Despite the surge in publications, the robustness and internal coherence of the findings differ sharply depending on which design, demographic, or cancer subtype one examines. This section critically evaluates the available clinical evidence, highlighting key patterns and challenges.

### 5.1. Epidemiological Studies

Several large epidemiological investigations have documented an inverse relationship between metformin prescription and the occurrence of several cancers, most notably breast, colorectal, prostate, and pancreatic tumors [28,39,40,41,42,43]. Aggregate data from subsequent meta-analyses point toward a dose–response effect in these findings, indicating that longer treatment spans and greater cumulative doses of the drug are linked to diminishing oncologic risk [44].

A number of large, population-based cohort investigations have documented a dose–risk gradient for metformin; extended therapy and greater cumulative exposure have both been associated with a lower incidence of several malignancies. That protective pattern, while statistically robust in many studies, does not hold uniformly across every cancer type or demographic group, making stratified subgroup analyses essential for drawing reliable conclusions [45,46].

Several biologically plausible mechanisms have been proposed to explain these epidemiologic observations, including enhanced insulin sensitivity, lowered systemic inflammation, and the alteration of key metabolic and growth signaling networks [7,47]. Additional studies have even associated metformin with better cancer-specific survival and longer progression-free intervals, hinting that its protective effects may extend beyond primary prevention alone [48].

However, as is often the case with observational data, these results remain correlational and may be distorted by variables such as glycemic control, co-administered drugs, and differential access to clinical services. The analysis falls short of proving cause-and-effect and offers little insight into cohorts that do not meet the diagnostic criteria for diabetes.

### 5.2. Retrospective Analyses

Retrospective studies that mine routine clinical databases frequently report that diabetic patients receiving metformin enjoy better overall survival (OS) and cancer-specific survival (CSS) than counterparts managed with insulin or sulfonylureas. This advantage appears robust across a spectrum of malignancies and treatment modalities, with several investigations noting that metformin may amplify the efficacy of concurrent chemotherapy or radiotherapy [49,50,51].

Subgroup analyses that sort patients by cancer stage, histological type, and treatment approach continue to reveal noteworthy differences in treatment response, implying that metformin might exert stronger benefits in certain clinical settings. In addition, multiple investigations have tested how metformin interacts with standard cancer therapies, observing possible synergy when the drug is combined with either chemotherapy or radiotherapy [6,51].

However, indication bias, fragmentary oncologic staging records, and the absence of standardized outcome metrics together constrict the interpretability and broader applicability of the findings. In addition, many published analyses overlook patient-specific insulin resistance profiles and the molecular subtypes intrinsic to the tumors themselves.

### 5.3. Prospective Clinical Trials

Prospective randomized controlled trials remain the definitive framework for assessing metformin’s potential anticancer activity. The following studies illustrate the diversity of the inquiry.

The McBETH Trial—registered as NCT02506630—is a phase II exploration that incorporated metformin into the neoadjuvant regimen for HER2-positive and triple-negative breast cancer. The protocol achieved a statistically heightened rate of pathologic complete response, yet the clinical gain was modest and not uniformly robust across the cohort [52].

The METEOR Trial, coded NCT01885013, paired metformin with chemotherapy in patients bearing HER2-negative breast malignancies. The investigators ultimately deemed the effects on progression-free survival inconclusive, leaving the primary question largely unanswered [53].

A separate study in metastatic pancreatic carcinoma, cross-referenced as NCT01101438, failed to yield any meaningful extension of overall or progression-free survival, undercutting the strong preclinical expectations that had predated it [54].

A recent meta-analysis by Wen et al. covering 22 distinct randomized trials drew several sobering conclusions. First, cancer-related mortality did not exhibit a statistically significant decline; second, progression-free survival benefits appeared confined to reproductive system tumors; and third, outcomes in certain digestive tract neoplasms actually worsened with metformin administration [55].

These mixed outcomes reflect the biological heterogeneity of tumors, non-uniform dosing regimens, and inadequate biomarker stratification in many trials (Table 3).

## 6. Biomarkers and Personalized Strategies in Metformin Oncology

Biomarker-guided clinical frameworks are emerging as a prerequisite for harnessing metformin’s anticancer promise [61,62]. Tumor metabolism, somatic alterations, and individual organic milieu each diverge sharply from case to case, complicating the task of patient selection. Recent clinical evidence highlights metformin’s efficacy as an adjuvant in metastatic breast cancer treatment, reinforcing the importance of patient-specific approaches [63]. Similarly, investigations in lung cancer point toward a nuanced interplay between metformin’s metabolic actions and tumor heterogeneity, suggesting that biomarker-driven strategies may be essential to unlock its full therapeutic benefit [64]. Extending this concept, immune-related data from phase 2 studies in prostate cancer indicate that metformin’s oncologic effects may also be mediated through modulation of host immunity, further underscoring the value of integrative biomarker frameworks for guiding therapy [65]. Research teams are now cataloging clues in four interlocking domains: DNA sequence variants, circulating metabolite signatures, volumetric imaging patterns, and integrative multi-omics assemblies.

Biomarker-guided clinical frameworks are emerging as a prerequisite for harnessing metformin’s anticancer promise [65]. Tumor metabolism, somatic alterations, and individual organic milieu each diverge sharply from case to case, complicating the task of patient selection. Research teams are now cataloging clues in four interlocking domains: DNA sequence variants, circulating metabolite signatures, volumetric imaging patterns, and integrative multi-omics assemblies [66,67,68]. A summary table classifying biomarkers by type and clinical relevance is provided in Table 4.

Table 3 summarizes various types of biomarkers that can predict the response to metformin therapy that aids in patient stratification, treatment selection, and the monitoring of treatment response in clinical practice.

Although a number of biomarkers appear promising for forecasting how well a patient will respond to metformin, their use in everyday clinical settings is still hampered by uneven validation, absent standard protocols, and sensitivity to specific study contexts. Future works, therefore, need to focus on biomarker-led clinical trials alongside integrated multi-omics platforms so that care teams can choose the right patients and track treatment effects in real time.

### 6.1. Critical Evaluation of Biomarker Use

Polymorphic variants within the uptake transporter OCT1 (genetic locus SLC22A1) have been a focal point in pharmacogenomic research on metformin [69]. Yet their ability to forecast treatment response in malignancies is hampered by uneven OCT1 expression across tumor types, functional overlap with OCT2 and MATE1, and mixed findings from the limited onco-clinical data set [70].

PI3K/AKT/mTOR alterations and individual AMPK SNPs indeed suggest working mechanisms, yet when evaluated alone, they rarely deliver precise clinical predictions. In a tumor context, hypoxia, local insulin resistance, and even patterns of immune cell infiltration often shape outcomes more decisively than any genotype [71].

Metabolic biomarkers like homeostasis model the assessment of insulin resistance (HOMA-IR) and the early-morning insulin concentration; they remain popular metabolic gauges for exploring the response gradient in non-diabetic cohorts. Even so, clinical utility is tethered by confounding variables such as habitual diet and body mass index, as well as the tests’ inherent lack of tumor specificity [72,73].

Multi-omics profiling provides a sweeping view of biological systems by integrating genomics, transcriptomics, proteomics, and metabolomics, yet it depends on a resilient bioinformatics backbone. Without platform standardization and prospective trial validation, only early-stage research remains.

### 6.2. Ongoing and Emerging Biomarker-Driven Trials

A number of clinical trials are now embedding biomarker analysis directly into their protocols, with the aim of refining patient selection and pinpointing the most effective therapies.

NCT01101438 focuses on breast cancer and specifically tests whether measurements of insulin sensitivity can anticipate the tumor shrinkage seen after pairing metformin with standard chemotherapy [74].

NCT05273099, set in early-stage endometrial cancer, uses molecular flags—such as PTEN and KRAS mutations—to decide which patients should receive metformin as an adjuvant treatment [75].

NCT02935309, centered on colorectal cancer, tracks circulating metabolic signatures to see if they can forecast treatment response among those already on the drug [76].

Taken together, these efforts signal a shift away from one-size-fits-all prescribing toward genuinely precision uses of metformin in the oncology clinic.

### 6.3. Future Directions

The precision use of metformin as an adjunct in oncology will hinge on several targeted research efforts.

One avenue is the assembly of multi-omics composite panels that marry genomic, metabolic, and imaging readouts into a single diagnostic lens. Another is the rigorous testing of tumor-specific predictive signatures within large, prospective studies that deliberately enrich relevant biomarkers, as well as adaptive trial frameworks that permit real-time adjustments based on patient response form a third priority.

Finally, serial liquid biopsy analyses—whether through circulating tumor DNA or metabolomic profiles—are essential for tracking biomarker changes as therapy unfolds.

A variety of proposed biomarkers have surfaced as indicators that might select patients for metformin treatment, yet clinical practice still lacks a single, routinized test. Research now hinges on creating unified validation workflows, reconciling different analytical platforms, and tailoring models to the specific tumor microenvironments involved. Only biomarker-driven trials, enrolling cohorts matched to the most promising profiles, will clarify when and for whom metformin emerges as a game-changing component of precision cancer care.

## 7. Long-Term Effects and Safety of Metformin in Cancer Therapy

Numerous cellular and molecular pathways respond to metformin, hinting that the drug could affect survival, wellbeing, and cancer return long after the first dose. Research has yet to agree, with results oscillating from solid support in breast malignancies to little benefit in prostate tumors (Table 5).

## 8. Ethical and Regulatory Challenges

The rediscovery of metformin as a potential anticancer drug is stirring a fresh round of regulatory debate. Off-label prescribing, adaptive-trial architecture, and the drug’s price-to-value calculus must all be settled before the molecule can enter the daily toolkit of oncologists in low-resource settings.

### 8.1. Regulatory Pathways for Repurposing

Repurposing metformin toward cancer treatment does not sidestep the usual regulatory hurdles. In the United States, the Food and Drug Administration permits sponsors to lean on pre-existing files via the 505(b)(2) route, a maneuver that trims both the calendar and the spending [81]. The European Medicines Agency, working in parallel, runs an Adaptive Pathways scheme that authorizes gradual patient entry while promoters fold in day-to-day evidence gathered after the first trial [82].

Even with those pathways in place, the regulatory apparatus insists on fresh, rigorous clinical trial evidence before a sponsor can extend a label into non-diabetic cohorts or into indications tied to distinct tumor types.

### 8.2. Ethical Implications of Off-Label Use

Metformin has carved out a niche in oncology, often prescribed off-label in research clinics that cannot wait for the usual regulatory rubber stamp [83]. Oncologists value the drug for its low cost and surprisingly rich laboratory backstory, even when the FDA or EMA has yet to rewrite their labels [84]. Informed consent must be more than a signature; patients should hear, in plain language, that the U.S. FDA and Europe’s EMA have yet to green-light metformin for solid tumors and that the evidence base remains provisional [85]. The problem of therapeutic misconception adds a second layer of difficulty. When the drug is paired with standard chemotherapy and presented in the familiar corridors of a teaching hospital, individuals may mistakenly assume that the off-label agent carries the same level of validation as their primary regimen [86,87]. Off-label prescribing is often the default in low- and middle-income contexts simply because the tablets are affordable. This price advantage, while practical, can blur ethical lines and create a two-tiered system where wealthier patients receive controlled trials, biomarker profiling, and close monitoring while poorer patients endure investigational use with little safety scaffolding [88].

Local institutional review boards and national health ministries carry the burden of closing those gaps. Rigid ethical safeguards—such as community-based consent processes, real-time adverse event logging, and independent data audits—must be woven into every protocol so that cost constraints do not silently redefine what constitutes acceptable care [89,90].

### 8.3. Trial Design Complexity and Global Economic Considerations

Testing a drug such as metformin for a new oncologic indication poses several methodological hurdles.

First, the protocol must enroll diabetics alongside non-diabetics without introducing confounding glucose control issues. Second, patient stratification may rely on circulating biomarkers, clinical chemistry, or even tumor genomics, each of which adds data-processing overheads. Trial success is not measured by overall survival alone; metrics like progression-free survival, quality-of-life shifts, and local recurrence rates start to dominate the clinical endpoint discussion.

Finally, managing an international, multi-site study inevitably stretches budgets and requires synchronizing ethics approvals, the shipment of investigational product, and real-time data interchange across disparate regulatory landscapes [91,92].

Cost-effectiveness modeling sits at the intersection of economics and public health planning; it asks whether a new treatment buys enough health for the money spent to warrant widespread adoption [93].

Quality-Adjusted Life Years, or QALYs, translate longevity into a utility scale by weighing each year lived by its quality. Early evidence suggests that metformin’s mild toxicity profile enables clinicians to rack up QALYs in high-risk, early-stage cancers [94].

Disability-Adjusted Life Years, often favored in low- and middle-income countries, flip the calculation by counting how many years of disability the intervention prevents. From that perspective, metformin shines as a front-line candidate in high-burden malignancies such as gastric, colorectal, and breast tumors [95].

An extra attraction lies in the drug’s low purchase price and the simplicity of swallowing a tablet. All the same, budget planners hesitate because the survival gains remain shaky outside trial populations, and reliable biomarkers to fine-tune eligibility have yet to materialize.

### 8.4. Promoting Ethical Global Access

A responsible rollout of metformin in cancer care hinges on equitable trial design and execution. Decision-makers might therefore draft repurposing policies that reward sponsors for enrolling underrepresented groups and for setting studies in low- and middle-income countries [81].

To bridge the funding gap, several agencies have turned to public–private partnerships that underwrite trial costs and guarantee affordable access once a drug secures its license [96].

At the finishing line, aligned global regulations—such as those outlined by the International Council for Harmonisation—could cut the busywork of duplicating protocols and help shift promising evidence into practice across borders.

## 9. Challenges in Integrating Metformin into Oncology

### 9.1. Challenges and Future Directions in Metformin Integration

Compelling preclinical studies and broad epidemiological correlations hint that metformin could play a role in cancer treatment, yet its routine adoption in oncology is far from settled. Obstacles include the drug’s pharmacokinetic variability across patient populations, its intricate biology, and the scarcity of large, forward-looking trials. The greater hurdle, however, lies in the complete absence of robust predictive biomarkers that could either enrich or exclude patients from metformin-based protocols. Until those scientific voids are filled, the agents promise will remain more hypothetical than practical (Table 6).

The meaningful integration of metformin into oncology hinges on closing wide gaps in its underlying biology, trial methodology, pharmacodynamics, and the tailoring of dosing to individual patient profiles. Achieving this conjunction will require an interdisciplinary consortium that fuses insights from oncology, pharmacology, molecular biology, and bioinformatics to delineate the drug’s exact niche in contemporary cancer treatment.

### 9.2. Multidisciplinary Imperatives

Bringing metformin into cancer care as a repurposed medication demands teamwork across several medical domains. Oncologists and clinical trial designers will have to mesh metabolic markers and genomic signatures with standard enrollment criteria. Pharmacists and dosing specialists are responsible for mapping new administration rhythms and cataloging every potential interaction with concurrent therapies. Bioinformaticians are essential for integrating omics data to produce real-time patient eligibility filters. Lastly, regulatory bodies should simplify the pathway for drugs that have already passed years of safety scrutiny.

### 9.3. Call to Action: Stakeholder-Specific Priorities

Metformin occupies a distinctive niche in the rapidly shifting field of precision oncology—affordable, mechanistically varied, and accessible almost everywhere. Nonetheless, its clinical repurposing has stalled because rigorous trials, biomarker-led patient selection, and dose refinement are still lacking. Moving forward will require a united effort from researchers, regulators, and industry partners to transform this well-tolerated metabolic drug into a practical weapon against cancer (Table 7).

## 10. Future Perspectives and Clinical Implications

Although promising preclinical studies and growing epidemiological signals support metformin’s anticancer potential, randomized controlled trials have yet to confirm a clear benefit across tumors. This discord probably arises from the following:

a The absence of biomarkers that guide patient enrollment;
b Heterogeneity among tumor types and clinical endpoints;
c Suboptimal dosing and pharmacokinetic adjustment in cancer care.

Future trials designed to clarify metformin’s therapeutic role should

a Target non-diabetic subjects as well as participants grouped by metabolic phenotypes;
b Incorporate predictive biomarkers such as OCT1 genotypes and insulin metrics;
c Define tumor-specific endpoints, including progression-free survival, recurrence rates, and quality-of-life measures;
d Embed mechanistic correlative studies that confirm on-target biological effects.

Because metformin is safe, inexpensive, and biologically plausible, it could still play a useful supportive role in precision cancer care, provided that studies enroll well-chosen biomarker-guided groups and follow a sound trial blueprint. This section outlines key directions and translational pathways for future development.

### 10.1. Personalized and Precision-Based Strategies

Because cancer cells differ from one another and because every patient has a unique metabolism, giving everyone the same dose of metformin no longer makes sense. Instead, combining the following allows for clinicians to spot patients most likely to improve and to tailor the drug to their biology:

a Molecular tumor profiling;
b Metabolic phenotyping;
c Genetic biomarkers (such as OCT1 variants and PI3K/AMPK mutations);
d Omics-guided patient stratification.

**Figure 3:**
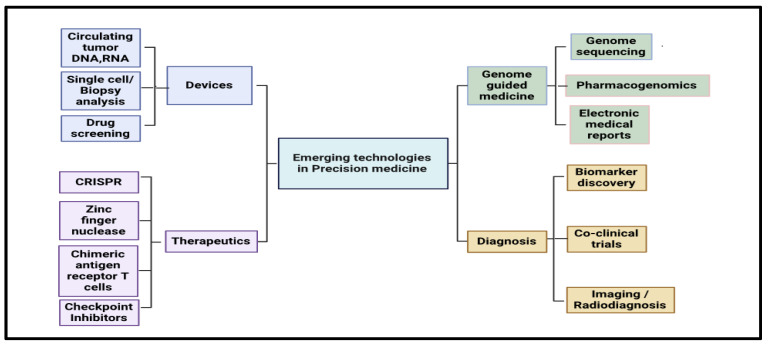
Concept map outlining emerging technologies in precision medicine, divided into four key domains: devices (including circulating tumor DNA/RNA analysis, biopsy-based diagnostics, and drug screening), therapeutics (such as CRISPR, zinc finger nucleases, CAR-T cell therapy, and checkpoint inhibitors), genome-guided medicine (featuring genome sequencing, pharmacogenomics, and electronic medical records), and diagnosis (covering biomarker discovery, co-clinical trials, and advanced imaging techniques).

**Figure 4:**
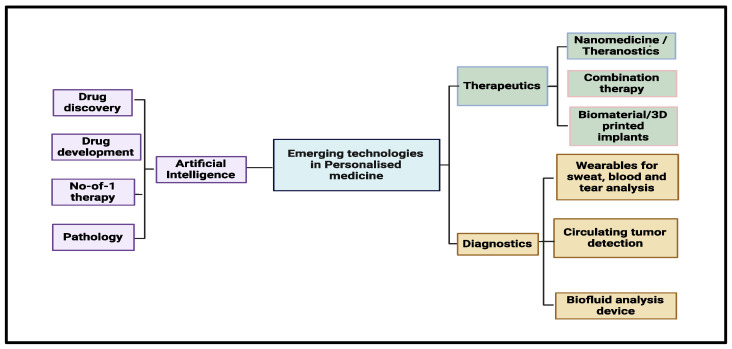
Mind map illustrating emerging technologies in personalized medicine, categorized into three major domains: artificial intelligence (including applications in drug discovery, development, pathology, and personalized No-of-1 therapy), therapeutics (such as nanomedicine, combination therapy, and 3D-printed biomaterials), and diagnostics (featuring innovations like wearables, circulating tumor detection, and biofluid analysis devices).

Future trials should use adaptive designs that adjust in real time to biomarker readings so that treatment can be fine-tuned (Figure 3 and Figure 4).

### 10.2. Novel Combinatorial Approaches

Metformin’s anticancer action might be strengthened when paired judiciously with other treatments, such as

a Chemotherapy: By imposing extra metabolic strain, it could render tumor cells more susceptible;
b Radiotherapy: The drug inhibits mitochondrial function, reduces hypoxia, and thus enhances radiosensitivity;
c Immunotherapy: Metformin appears to boost immune cell entry and shrink immunosuppressive populations;
d Targeted therapies: It may work synergistically with drugs that block mTOR, IGF-1R, or angiogenesis.

Although preclinical studies back these pairings, proving them safe and effective in patients remains crucial.

### 10.3. Drug Repurposing as a Broader Paradigm

Metformin exemplifies the potential of drug repurposing in oncology, offering a time- and cost-efficient alternative to de novo drug development. Success in this domain can encourage the exploration of other off-patent agents with untapped oncologic applications.

Policy-level support, including regulatory incentives and dedicated funding mechanisms, will be essential to overcome commercial disincentives that often impede repurposing efforts.

### 10.4. Clinical Trial Priorities

Upcoming clinical trials ought to fill existing knowledge gaps by concentrating on

a Non-diabetic study groups, in which mechanistic actions can be seen apart from blood sugar control;
b Biomarker-driven cohorts, using molecular or metabolic signatures to predict who is likely to respond;
c Tumor-specific endpoints, such as progression-free survival, rates of recurrence, and treatment-related harms;
d Patient-reported metrics, including fatigue, appetite, and overall quality of life, to gauge wider clinical value;
e Cross-disciplinary teamwork among oncologists, endocrinologists, molecular biologists, and trial designers will be vital to creating effective studies.

### 10.5. Implications for Clinical Practice

Metformin is still a medicine being trialed in oncology, yet certain patient profiles hint at real utility. Oncologists already see diabetic patients who tolerate the drug, extending its use during chemotherapy, sometimes watching tumors respond in ways that historic trials do not fully explain. The observation is informal, almost anecdotal, but it weighs on clinical practice.

Separate from the diabetes label, doctors encounter middle-aged people with metabolic syndrome or stubborn insulin resistance. This non-diabetic group does not fit neatly into existing protocols, yet their insulin profiles provoke questions about whether they, too, can gain an edge. Trials targeting them have yet to start, but researchers sketch preliminary inclusion criteria on whiteboards and hospital projections.

High-risk subsets such as severely obese individuals or patients harboring pre-malignant tissue invite an even earlier testing horizon. Some cancer centers already screen for these phenotypes, mapping participants who might enter the next wave of prevention trials.

Even so, selection remains delicate. Renal clearance limits, gastrointestinal side effects, and rare but serious lactic acidosis incidents cannot be brushed aside, particularly with patients who have no diabetes label.

For the moment, metformin rests at an unusual crossroads where oncology meets metabolic physiology and bespoke medicine. Its low cost and long history compel investigators to ask whether a familiar compound can disrupt drug development norms. The answer, shaped by pent-up mechanistic clues and evolving biomarker panels, will decide whether metformin earns a permanent seat in twenty-first century treatment algorithms.

## 11. Conclusions

Metformin occupies a distinctive niche in precision oncology, where affordability and biological plausibility converge. Evidence demonstrates that it modulates cellular metabolism, oncogenic signaling, cancer stemness, and immune surveillance. While preclinical and epidemiological studies consistently support its anticancer potential, clinical outcomes remain inconsistent, largely due to tumor heterogeneity, non-uniform dosing, and the absence of biomarker-driven patient selection.

Moving forward, the integration of genomic, metabolic, and multi-omics biomarkers into trial designs is essential to identify responsive subgroups. Future studies should prioritize tumor-specific, biomarker-enriched randomized trials with well-defined clinical endpoints. If validated in such settings, metformin could transition from a repurposed adjunct to a cost-effective and accessible component of precision oncology, bridging the gap between mechanistic promise and real-world cancer care.

## Figures and Tables

**Table 1:** Canonical vs. non-canonical mechanisms of metformin in cancer.

Canonical Pathways (AMPK-Dependent)	Non-Canonical Pathways (AMPK-Independent)
Activation of AMPK (energy stress sensor)	Inhibition of mitochondrial complex I
Inhibition of mTOR signaling → ↓ protein synthesis, cell growth	Altered NAD^+^/NADH ratio and redox status
Inhibition of hepatic gluconeogenesis → ↓ insulin/IGF-1 signaling	Modulation of microRNA expression
Suppression of cancer cell proliferation via cell cycle arrest	Disruption of cancer stem cell dynamics
Enhanced immune response via T cell activation and PD-L1 downregulation	Epigenetic remodeling and chromatin accessibility changes

Arrows: decreased.

**Table 2:** Preclinical evidence supporting the anticancer effects of metformin.

Study Reference	Category	Cancer Type	Key Findings
Hua et al. [27]	In Vitro Studies	Various	Metformin modulates cancer hallmarks via AMPK activation and insulin pathway inhibition.
Yu et al. [28]	In Vitro Studies	Various	Observational studies showed benefit; RCTs did not confirm reduction in cancer outcomes.
Mu et al. [29]	In Vitro Studies	Various	Activated AMPK, inhibited proliferation, and promoted apoptosis in cancer cells.
Sanati et al. [30]	In Vitro Studies	Glioblastoma multiforme	Reviewed metformin’s anticancer potential against GBM.
Puła et al. [31]	In Vitro Studies	Various	Suggested preventive and therapeutic potential of metformin in neoplasms.
Mostafavi et al. [32]	In Vivo Studies	Solid tumors	Metformin reprograms CAFs and impairs tumor-supportive environment.
Cirillo et al. [33]	Mechanistic Studies	Breast	Metformin inhibits the PI3K/AKT pathway and metastasis-related CXCR4 expression.
Zhang et al. [34]	Mechanistic Studies	Multiple	Meta-analysis confirmed tumor burden reduction across cancer types.

**Table 3:** Summary of the current clinical trials evaluating the effectiveness of metformin in the cancer treatment.

Reference	Study Type	Cancer Type	Key Findings	Limitations	Clinical Implications
Dickerman et al. [56]	Target trial emulation	Multiple cancers	No significant effect on cancer incidence	Observational design constraints	Limited role in prevention
Kumar et al. [57]	RCT (neoadjuvant chemo + metformin)	Breast (HER2+, TNBC)	Higher pathological response rates with metformin	Small sample, open-label	Possible synergy with chemotherapy
Papadakos et al. [58]	Preclinical + early clinical	Hepatocellular carcinoma	Suggests benefit with immunotherapy	Lack of standardized dosing	Potential role in HCC; needs further trials
Kassem et al. [59]	RCT (non-diabetic breast cancer)	Breast	Reduced chemotherapy-related toxicities (neuropathy, mucositis, fatigue)	Open-label, no biomarker validation	Supportive role to reduce toxicity
Kennedy et al. [60]	RCT (pembrolizumab ± metformin)	Melanoma	No survival benefit observed	Small metformin subgroup	Limited effect in immunotherapy setting
Wen et al. [61]	Meta-analysis (22 RCTs)	Multiple cancers	No OS benefit; modest PFS benefit in reproductive cancers	Heterogeneity across trials	Benefits may be tumor-type-specific

**Table 4:** Classification of predictive biomarkers into various categories.

Biomarker Type	Details	Examples	Predictive Utility	Limitations
Genetic	Influence metformin transport, metabolism, and pathway interaction	*OCT1 (SLC22A1)* polymorphisms, *PI3K/AKT/mTOR* mutations, *PTEN* loss, AMPK SNPs	Predict drug uptake; insulin pathway sensitivity	Inconsistent translation across tumor types; limited specificity; transporter expression varies by tissue and tumor context
Metabolic	Reflect systemic/tumor metabolic state and insulin sensitivity	Fasting insulin, glucose, HOMA-IR, metabolomic signatures	May indicate response to metabolic stress or AMPK activation	Confounded by comorbidities (e.g., obesity, T2DM); not tumor-specific
Imaging	Monitor real-time metabolic/structural tumor adaptation	*18F-FDG PET*, DCE-MRI, diffusion-weighted MRI	Non-invasive, dynamic assessment	Variability in resolution and interpretation; lacks mechanistic depth
Multi-Omics	Layered profiling for comprehensive tumor biology	Genomic + transcriptomic + proteomic + metabolomic integration	Improves patient stratification and therapeutic targeting	High cost; complex data interpretation; not yet standardized

**Table 5:** Summary of clinical outcomes, mechanistic insights, and safety considerations for metformin in oncology.

Domain	Key Findings	Evidence Level	Remarks	Key Reference
Overall Survival (OS)	Improved OS in metformin users across cancers (e.g., breast, lung, prostate); reduced cancer-related mortality in diabetics	Moderate (meta-analyses, observational studies)	Needs validation from randomized controlled trials (RCTs)	Yang J et al. [77]
Safety Profile	Generally well tolerated; GI side effects most common; rare lactic acidosis in high-risk patients	High (clinical practice data)	Risk stratification essential for non-diabetic populations	UK NICE Guidelines [78]
Contraindications	eGFR < 30 mL/min/1.73 m^2^; severe hepatic or cardiac dysfunction; respiratory failure; hypersensitivity history	High (established clinical guidelines)	Pretreatment screening is mandatory	UK MHRA [79]
Drug Interactions	May interact with chemotherapy and immunotherapy; potential for altered pharmacokinetics and immune-related side effects	Moderate (emerging data from clinical settings)	Monitor closely when combined with novel agents	Heckman-Stoddard BM et al. [80]

**Table 6:** Challenges in integrating metformin into oncology and proposed solutions.

Challenge	Description	Proposed Solution
Patient Heterogeneity	Variability in tumor type, disease stage, metabolic status, and genetic background affects response to metformin.	Adopt biomarker-guided, stratified trial designs to identify responsive subgroups.
Tumor Type and Biological Complexity	Efficacy varies across cancers due to differing oncogenic drivers and metabolic profiles.	Conduct tumor-specific studies and mechanistic research to define indications.
Biological Redundancy and Resistance Mechanisms	Cancer cells may bypass AMPK/mTOR inhibition via alternate metabolic or signaling pathways.	Design combination regimens that target compensatory mechanisms.
Pharmacokinetic and Dosing Variability	Standard antidiabetic dosing may not ensure therapeutic levels for antitumor effects.	Perform oncology-specific pharmacokinetic and dose optimization studies.
Drug Interactions and Treatment Integration	Metformin may alter efficacy or toxicity profiles of concurrent chemotherapy or immunotherapy.	Design rational combination trials with integrated pharmacovigilance.
Lack of Dedicated Oncology Trials	Most data are derived from retrospective or diabetic cohorts, limiting applicability.	Launch well-powered RCTs in non-diabetic patients with tumor-specific endpoints.

**Table 7:** Translational call to action for metformin repurposing in oncology.

Stakeholder	Priority Actions
Researchers	Establish reliable biological markers that reliably predict patient sensitivity to metformin. Pursue laboratory investigations using cancer stem cell models and platforms that integrate immunometabolic pathways. Systematically test whether metformin enhances the efficacy of paired modalities such as immune checkpoint inhibitors or focused radiotherapy.
Clinicians	Clinicians should exercise restraint when prescribing off-label, favoring indications supported by rigorous clinical trials. Biomarker assessment, when accessible, can direct therapy toward the patients most likely to benefit. In non-diabetic cohorts, vigilant monitoring of metabolic indices and known contraindications remains essential to prevent avoidable harm.
Regulators and Policymakers	Encourage the use of adaptable regulatory routes such as the FDA 505(b)(2) pathway and the EMA’s Adaptive Pathways. Increase public–private funding for studies that test old drugs in new settings. Weave QALY and DALY metrics into pricing and access rules from the start.
Global Health Stakeholders	Strengthen fair access in low- and middle-income countries by using pooled purchasing. Include these nations early on when drafting clinical protocols. Prevent misuse by banning off-label applications that lack proper review.

## Data Availability

Available upon request.
